# Comparison of cognitive and UHDRS measures in monitoring disease progression in Huntington’s disease: a 12-month longitudinal study

**DOI:** 10.1186/2047-9158-3-15

**Published:** 2014-07-12

**Authors:** Eng A Toh, Michael R MacAskill, John C Dalrymple-Alford, Daniel J Myall, Leslie Livingston, Sandy AD Macleod, Tim J Anderson

**Affiliations:** 1 Department of Medicine, Christchurch School of Medicine & Health Sciences, University of Otago, Christchurch, New Zealand; 2 New Zealand Brain Research Institute, Christchurch, New Zealand; 3 Department of Psychology, University of Canterbury, Christchurch, New Zealand; 4 Brain Injury Rehabilitation Service, Canterbury District Health Board, Christchurch, New Zealand; 5 Department of Neurology, Canterbury District Health Board, Christchurch, New Zealand

**Keywords:** Huntington’s disease, Disease progression, Cognition, UHDRS, Longitudinal

## Abstract

Progressive cognitive decline is a feature of Huntington’s disease (HD), an inherited neurodegenerative movement disorder. Comprehensive neuropsychological testing is the ‘gold standard’ to establish cognitive status but is often impractical in time-constrained clinics. The study evaluated the utility of brief cognitive tests (MMSE and MoCA), UHDRS measures and a comprehensive neuropsychological tests battery in monitoring short-term disease progression in HD. Twenty-two manifest HD patients and 22 matched controls were assessed at baseline and 12-month. A linear mixed-effect model showed that although the HD group had minimal change in overall global cognition after 12 months, they did show a significant decline relative to the control group. The controls exhibited a practice effect in most of the cognitive domain scores over time. Cognitive decline at 12-month in HD was found in the executive function domain but the effect of this on global cognitive score was masked by the improvement in their language domain score. The varying practice effects by cognitive domain with repeated testing indicates the importance of comparing HD patients to control group in research trials and that cognitive progression over 12 months in HD should not be judged by changes in global cognitive score. The three brief cognitive tests effectively described cognition of HD patients on cross-sectional analysis. The UHDRS cognitive component, which focuses on testing executive function and had low variance over time, is a more reliable brief substitute for comprehensive neuropsychological testing than MMSE and MoCA in monitoring cognitive changes in HD patients after 12 months.

## Background

Huntington’s disease (HD) is an inherited neurodegenerative disease caused by expansion of CAG trinucleotide repeats secondary to mutation in the huntingtin gene on chromosome 4p16.3 [1]. The disease is characterized by involuntary hyperkinetic movements, cognitive impairment and behavioural disorders. Cognitive impairment, which may be evident even in gene-positive individuals yet to be clinically diagnosed [2-5], is progressive in nature and a contributing factor in the loss of everyday function [6]. Subtle cognitive impairment can be overlooked by clinicians during routine follow-up [7], indicating the need for easily administered and yet robust tool to detect cognitive changes in HD. Comprehensive neuropsychological testing is necessary to verify cognitive status. However, neuropsychological batteries are time-consuming and brief cognitive screening tools such as the Mini Mental State Examination (MMSE) and the Montreal Cognitive Assessment (MoCA) are commonly used in clinical settings and in a broad range of conditions. The Unified Huntington’s Disease Rating Scale (UHDRS), a standard assessment tool for HD, also includes a brief cognitive component.

The MMSE [8] comprises eleven questions spanning five aspects of cognitive function: executive function, language, memory function, visuospatial ability and orientation. It has good inter-rater, test and re-test reliability in differentiating cognitive status in dementia syndromes [9] and other disorders featuring cognitive impairment [10]. Nevertheless, it is influenced by demographic factors such as age, education and cultural background [9,11,12]. The MoCA places greater emphasis than the MMSE on naming, attention, abstraction and delayed recall, functions that are most likely to be compromised in the earlier stages of cognitive impairment and unlike the MMSE, it compensates for education level [13]. Both the MMSE and MoCA have been employed as measures of cognitive performance in manifest HD patients [14-17] and MoCA was also found to have higher sensitivity without losing specificity than the MMSE in identifying those with cognitive impairment in HD [16]. Furthermore, Bezdicek et al. [17] demonstrated a strong correlation between the MoCA scores and comprehensive neuropsychological assessment scores in manifest HD patients. The UHDRS cognitive component [18] includes three tests of executive function – letter fluency test, Symbol Digit Modalities test and Stroop test; which can be used with corrected norms to attenuate the impact of various demographic variables [19,20].

The progressive nature of HD means that any cognitive assessments should also be useful longitudinally. HD patients are routinely followed up at clinics at 6-month and 12-month intervals thus it is preferable that brief cognitive assessment tools are sensitive to changes even over relatively short time intervals. Effective yet brief cognitive tools would enable easier detection of cognitive changes in HD patients in clinic settings than time consuming comprehensive cognitive assessment and also assist health care providers in designing treatment and care plans aimed at improving patient’s quality of life. MMSE and MoCA have been extensively evaluated in previous cross-sectional HD studies [14-16] but to our knowledge, there is no longitudinal data on the utility of these brief cognitive tests compared to UHDRS cognitive assessment in monitoring cognitive changes in HD patients. Therefore the objective of this study was to examine and compare the relative utility of two widely used brief cognitive tests (MMSE and MoCA) concurrently with the UHDRS cognitive assessment to a comprehensive neuropsychological test battery for monitoring cognitive changes in HD patients over a short interval of 12 months. Such a direct comparison has not been previously reported.

## Methods

Study participants were a convenience sample of 22 manifest HD patients (10 males and 12 females) with mild to moderate disease severity and 22 age, gender and education matched control volunteers recruited through the New Zealand Brain Research Institute database (Table 1). Patients were genetically verified and clinically diagnosed by a movement disorders specialist (TJA). Participants identified themselves as native speakers of English and consented to participate in compliance with the requirements of the New Zealand Ministry of Health Ethics Committee.

**Table 1:** Demographic characteristics (mean and SD) of control and HD groups

	**Mean (SD)**	
	**Control group**	**HD group**
	**n = 22**	**n = 22**
**Age**	50 (15)	50 (15)
**CAG repeat no.**	-	44 (4)
**Education level (years)**	13 (2)	13 (2)
**Follow-up interval (weeks)**	53 (3)	54 (4)
**Retention rate at 12-month**	91% (n = 20)	100% (n = 22)

### Experiment procedure

The MMSE, MoCA and a comprehensive neuropsychological test battery were administered to all participants. The comprehensive assessment of cognitive function used 19 neuropsychological tests to assess six domains of cognitive function (executive function; working memory and attention; learning and memory; processing speed; language; and visuospatial function). These tests were: executive function: letter, action and category fluency tests [21], Trail Making Test (Part B), Stroop-interference test; working memory and attention: digits forward, backward and sequencing tests [22], Symbol Digit Modalities Test [23] and Ruff 2 & 7 Cancellation Test – Accuracy [24]; learning and memory: Short California Verbal Learning Test-II and Brief Visuospatial Memory Test-Revised; processing speed: Stroop-word reading, Stroop-colour naming, Trail Making Test (Part A) and Ruff 2 & 7 Cancellation Test – Speed; language: Brief Boston Naming Test [25] and Indiana University Token Test [26]; and visuospatial function: Judgement of Line Orientation test (Form H) and Rey Complex Figure Copying test. For the MMSE, both alternatives (‘World’ spelled backwards and serial sevens) were assessed. The number of tests administered was evenly distributed over two separate sessions, one week apart and presented in the same order for all participants. Each session began with the MMSE in the first and MoCA in the second session. The three-part UHDRS, comprising motor, cognitive and behavioural components, was administered in the first session to the HD group only. All returning participants were reassessed in identical manner 12 months later.

### Data analysis

Cognitive status – normal, mild cognitive impairment (MCI) or dementia – of HD participants was determined using evidence from the neuropsychological test battery and the UHDRS. Criteria for mild cognitive impairment (MCI) followed that described for Parkinson’s disease by Dalrymple-Alford et al. [27], with a requirement of 2 measures at -1.5SD or equivalent within a single domain; and dementia criteria followed that of Peavy et al. [5], which defined HD dementia as having cognitive deficits in at least two areas of cognition not limited to memory deficits in the context of impaired everyday function as determined through the UHDRS Functional Independence Scale.

The raw score of each component test in the neuropsychological test battery was converted to a standard z-score using test-specific norms so that objective comparison can be made across component tests, regardless of individual scale ranges and distributions. Domain-specific scores were mean aggregated scores of component tests within a cognitive domain and the average scores across all six cognitive domains determined the global cognitive z-score (overall global cognition). The MoCA scores were adjusted to participants’ education level [13]. The three cognitive tests (letter fluency, SDMT and Stroop tests) in the UHDRS cognitive component were part of the neuropsychological test battery so the mean aggregate z-score of these tests was used as the UHDRS cognitive score for both the HD and control groups.

### Statistical analysis

For each of the measures, the differences between groups at baseline and the change over 12 months were determined using linear mixed-effects models [28]. These models take into account the correlated measurements within a participant when assessing the differences between groups and changes over time. The relationship between global cognition and brief cognitive tests, baseline and 12-month scores were assessed using *R*^*2*^ correlation coefficient from simple linear models. Bootstrapping [29] was used to assess differences between *R*^*2*^ correlation coefficients as standard analytical techniques were not applicable. Bootstrapping involved the original sample being resampled with replacement and the difference between *R*^*2*^ values in this new sample being determined. This was repeated 1000 times and the resulting distribution of differences in *R*^*2*^ values gave an indication of the mean difference and a 95% confidence interval. Cohen’s *d* was used to report effect sizes of differences between groups.

## Results

### Cognition at baseline and change over time

**Figure 1:**
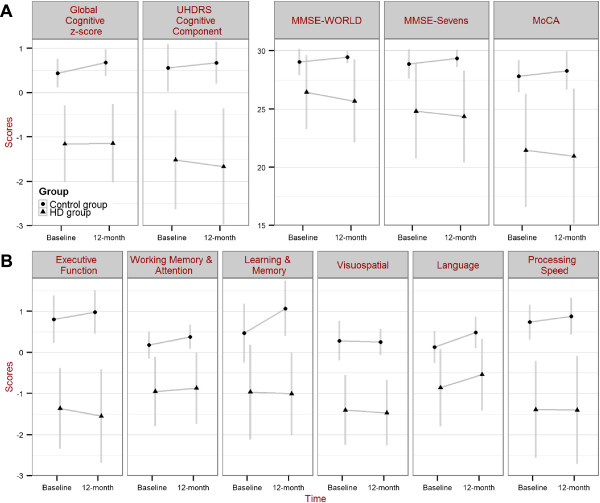
**Change in cognitive scores over 12 months.** Baseline and 12-month scores for control and HD groups in: **(A)** overall global cognition; UHDRS cognitive assessment; MMSE-WORLD; MMSE-Sevens; MoCA; and **(B)** the six cognitive domains. Group mean and SD are shown.

At baseline, six HD patients had normal cognition, 10 met criteria for MCI, and six had dementia [5]. All 22 controls had normal cognition. The HD group showed significantly reduced scores (*t* > 3.5, *p* ≤ 0.001) in overall global cognition and brief cognitive tests compared to controls both at baseline and at 12-month follow-up. The mean effect sizes for the two years combined in overall global cognition was *d* = 2.6 whereas in the brief cognitive tests, they ranged from *d* = 1.3 in the MMSE with ‘World’ spelled backwards to *d* = 2.4 in UHDRS cognitive assessment (Figure 1A, Table 2). In terms of domain-specific scores, the HD group had significantly lower scores (*t* > 4.7, *p* < 0.001) compared to controls across all cognitive domains and the mean effect sizes for baseline and 12-month combined ranged from the smallest (*d* = 1.5) in the language domain to the largest (*d* = 2.8) in the executive function domain (Figure 1B, Table 2). Individual component test scores in HD and control groups at baseline and 12-month are detailed in Additional file 1: Figure S1 and brief cognitive tests in Additional file 2: Table S1.

**Table 2:** **Scores**^**# **^**at baseline, within-group changes and group over time interactions of control and HD groups**

**Measures**	**z-scores**				
	**Control group**		**HD group**		**HD vs. Controls**
	**BaselineMean [95% CI]**	**Change after 12 monthsMean [95% CI]**	**BaselineMean [95% CI]**	**Change after 12 monthsMean [95% CI]**	**Relative change after 12 monthsMean [95% CI]**
**Overall global cognition**	0.4 [0.2 – 0.7]	0.2 [0.1 – 0.3], *p* < 0.001	-1.2 [-1.4 – -0.9]	0.01 [-0.1 – 0.1], *p* = 0.8	-0.2 [-0.4 – -0.1], *p* = 0.006
Executive function	0.8 [0.4 – 1.2]	0.1[-0.04 – 0.1], *p* = 0.1	-1.4 [-1.7 – -1.0]	-0.2 [-0.4 - -0.02], *p* = 0.03	-0.3 [-0.6 – -0.1], *p* = 0.01
Working memory & attention	0.2 [-0.1 – 0.5]	0.2 [0.05 – 0.3], *p* = 0.01	-1.0 [-1.2 – -0.7]	0.08 [-0.1 – 0.2], *p* = 0.3	-0.1 [-0.3 – 0.1], *p* = 0.3
Learning & memory	0.5 [0.1 – 0.9]	0.6 [0.3 – 0.9], *p* < 0.001	-1.0 [-1.4 – -0.6]	-0.04 [-0.3 – 0.2], *p* = 0.8	-0.6 [-1.0 – -0.3], *p* < 0.001
Processing speed	0.7 [0.3 – 1.1]	0.1 [0.01 – 0.03], *p* = 0.04	-1.4 [-1.8 – -1.0]	-0.01 [-0.1 – 0.1] *p* = 0.8	-0.2 [-0.3 – 0.02], *p* = 0.09
Language	0.1 [-0.2 – 0.4]	0.3 [0.1 – 0.6], *p* = 0.008	-0.9 [-1.2 – -0.6]	0.3 [0.1 – 0.6], *p* = 0.01	-0.02 [-0.4 – 0.3], *p* = 0.9
Visuospatial	0.3 [0.0 – 0.6]	-0.02 [-0.2 – 0.2], *p* = 0.8	-1.4 [-1.7 – -1.1]	-0.07 [-0.2 – 0.3], *p* = 0.5	-0.04 [-0.3 – 0.2], *p* = 0.8
**UHDRS cognitive score**	0.6 [0.2 – 1.0]	0.06 [-0.1 – 0.2], *p* = 0.5	-1.5 [-1.9 – -1.1]	-0.2 [-0.3 - -0.001], *p* = 0.048	-0.2 [-0.4 – 0.01], *p* = 0.06
**Measures**	**Points**				
	**Control group**		**HD group**		**HD vs. Controls**
	**BaselineMean [95% CI]**	**Change after 12 monthsMean [95% CI]**	**BaselineMean [95% CI]**	**Change after 12 monthsMean [95% CI]**	**Relative change after 12 monthsMean [95% CI]**
**MMSE**					
with WORLD item	29 [28 – 30]	0.4 [-0.3 – 1.1], *p* = 0.3	26 [25 – 28]	-0.8 [-1.5 – 0.08], *p* = 0.03	-1.2 [-2.2 – -0.2], *p* = 0.02
with Sevens item	29 [28 – 30]	0.5 [-0.4 – 1.4], *p* = 0.3	25 [24 – 26]	-0.5 [-1.3 – 0.4], *p* = 0.3	-1.0 [-2.2 – 0.3], *p* = 0.1
**MoCA**	28 [26 – 30]	0.4 [-0.6 – 1.4], *p* = 0.5	21 [20 – 23]	-0.5 [-1.5 – 0.5], *p* = 0.3	-0.9 [-2.3 – 0.5], *p* = 0.2
UHDRS motor score*	-	-	42 [33 – 51]	7.4 [3.4 – 11.3], *p* < 0.001	-
UHDRS behavioural score*	-	-	23 [17 – 30]	0.7 [-6.4 – 7.8], *p* = 0.8	-

*UHDRS motor and behavioural components were assessed in the HD group only.

^#^Baseline and 12-month SD and ranges for all variables are detailed in Additional file 2: Table S1 and score changes after 12 months in individual component tests are shown in Additional file 1: Figure S1.

There was an overall pattern of improvement in the control group across all cognitive tests after 12 months. In contrast, the HD group showed minimal change in their global cognitive z-score and a general decline across all brief cognitive tests scores after 12 months, for which it was statistically significant (*t* < - 2.0, *p* = 0.048) in UHDRS cognitive score and MMSE with ‘World’ spelt backwards (Figure 1A, Table 2). The control group exhibited an increase in score at 12 months in most cognitive domains (*t* > 2.2, *p* < 0.04) excepting executive function and visuospatial domains (*t* < 1.6, *p* > 0.1). Contrastingly, the HD group demonstrated a decline (*t* = 2.3, *p* = 0.03) in executive function domain but an increase (*t* = 2.7, *p* = 0.01) in language domain z-score 12 months later. There were no significant absolute changes (*t* < 1.2, *p* > 0.3) in the other cognitive domains in the HD group (Figure 1B, Additional file 2: Figure S1). A relative change over time (i.e. relative deterioration) in the HD group compared to change in scores in the control group was significant for global cognitive z-score, MMSE with ‘World’ spelled backwards, executive function, and learning and memory domain scores (Table 2). There was a significant worsening (*t* = 3.9, *p* < 0.001) of the UHDRS motor score over 12 months in the HD group but no change in behavioural score (Table 2).

### Usefulness of scores for measuring change over time

There are several considerations to take into account when determining which brief cognitive test has greatest utility for measuring cognition over time. This includes how well the score reflects overall global cognition, whether there is any ceiling effect, and how noisy (variability of score residuals) the score is after taking systematic changes into consideration.

**Figure 2:**
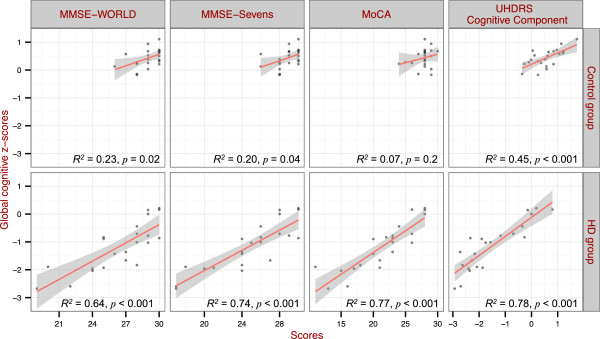
**Correlations between brief cognitive screening tests scores and global cognitive z-scores at baseline.** The control group is shown in the top row and the HD group in the bottom row. The *R*^*2*^ and the *p* values are shown for each of the brief cognitive screening tests.

Simple linear models confirmed that scores of all three brief cognitive screening tests, as judged by their *R*^*2*^ values, were significantly correlated with the scores of the full neuropsychological test battery at baseline (Figure 2) and 12-month (not shown). Bootstrap procedures confirmed that there were no significant differences between the three brief cognitive screening tests in extent of correlation with overall global cognition (Table 3). Thus all brief cognitive tests provided a reasonable cross-sectional measure of global cognition.

**Table 3:** **Comparison of ***R*^*2*^**differences of relationships between brief cognitive tests and full cognitive battery**

	**Differences in *R***^***2 ***^**values [95% CI]**		
	**MMSE – WORLD**	**MMSE - Sevens**	**MoCA**
**UHDRS cognitive component**	0.1 [-0.0 – 0.4]	0.1 [-0.1 – 0.3]	0.0 [-0.2 – 0.2]
**MMSE - WORLD**	-	-0.1 [-0.3 – 0.1]	-0.1 [-0.4 – 0.0]
**MMSE - Sevens**	-	-	-0.0 [-0.2 – 0.1]

**Figure 3:**
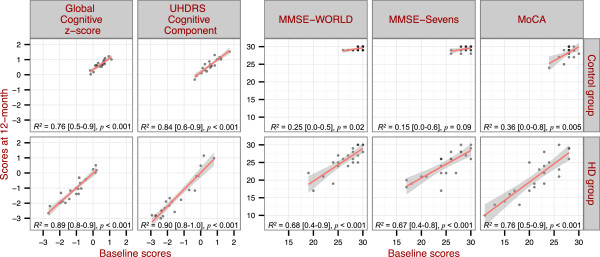
**Correlations between baseline and 12-month scores of the five cognitive measures.** Control group is shown in the top row and HD group in the bottom row. The *R*^*2*^, 95% CI (in square brackets) and *p* values of the relationship are shown for each of the cognitive measures.

To determine the variability of score residuals over time, simple linear models were fitted to the baseline and 12-month scores of overall global cognition and brief cognitive tests. The correlations within a test over time were evaluated by examining the *R*^*2*^ values of the model fits (Figure 3). In the control group, the range of scores in MMSE and MoCA was narrow due to a ceiling effect hence contributing to *R*^*2*^ values (*R*^*2*^ < 0.36). In contrast, the global z-score and UHDRS cognitive component showed greater utility in the control group, with a wider range of values together and small deviations from the linear fit, resulting in high *R*^*2*^ values. In the HD group the baseline scores were well correlated (*R*^*2*^ > 0.67) with 12-month scores for overall global cognition and all three brief cognitive tests (Figure 3). The comprehensive neuropsychological test battery and UHDRS cognitive component, as confirmed by bootstrap procedures, had smaller deviations from the linear fit than the two versions of the MMSE but not the MoCA (Table 4).This finding indicates that the two versions of MMSE had higher measurement noise (i.e. greater score variability over time), compared to overall global cognition and UHDRS cognitive assessment after 12 months.

**Table 4:** **Comparison of ***R*^*2*^**differences of relationships between baseline and 12-month scores in cognitive measures**

	**Differences in *R***^***2 ***^**values [95% CI]**			
	**UHDRS cognitive component**	**MMSE - WORLD**	**MMSE - Sevens**	**MoCA**
**Global cognitive z-score**	-0.0 [-0.1 – 0.1]	0.2 [0.0 – 0.4]*	0.2 [0.0 – 0.5]*	0.1 [-0.0 – 0.3]
**UHDRS cognitive component**	-	0.2 [0.0 – 0.5]*	0.2 [0.0 – 0.4]*	-0.1 [-0.0 – 0.4]
**MMSE - WORLD**	-	-	0.0 [-0.2 – 0.1]	-0.1 [-0.3 – 0.1]
**MMSE - Sevens**	-	-	-	-0.1 [-0.3 – 0.2]

*The estimated 95% CI of *R*^2^ differences is greater than ‘0.0’ which suggests that the difference in *R*^2^ values between the two cognitive measures tested could be considered significant.

In summary, the combination of showing a significant decline of cognition in HD (Table 2), high correlation with global cognitive z-score (Figure 2), and lower variance of score residuals over time (Figure 3) compared to other brief cognitive assessments, indicated the UHDRS cognitive component performed the best of the brief cognitive tests in assessing and monitoring cognition in HD patients over a 12-month period.

## Discussion

This study attempted to evaluate the usefulness of two widely used brief cognitive assessment tools (MMSE and MoCA) simultaneously with UHDRS cognitive component for monitoring cognitive changes in manifest HD patients over a 12-month interval by comparing them to a comprehensive neuropsychological test battery. In the process of evaluating the usefulness of these brief cognitive tests, we demonstrated that there was no significant change in overall global cognition in the presence of significant decline in the executive function domain in manifest HD patients after 12 months. Relative to the control group, which showed an increase in overall global cognitive z-score and learning and memory domain score over a 12-month period, there was significantly less change in domain-specific scores in the HD group over that period. The MMSE and MoCA were less effective than the UHDRS cognitive assessment for monitoring cognitive changes in manifest HD patients over 12 months.

### Domain-specific cognitive performance

Cognitive decline, which has been shown to assume a relatively slow course especially in the early stages of HD [30-33], is a well-established hallmark of HD. Overall, our findings corroborated with other longitudinal studies on pre-manifest and early manifest HD patients wherein, relative to a control group, cognitive decline were evident after a 12-month interval in the HD group [30] and, similar conclusions were made at 24-month follow-up [31]. The significant decline in executive function domain score in the HD score was consistent to a study by Bachoud-Lévi et al. [32], which demonstrated that cognitive deterioration in early stages patients is limited to attention and executive functions. However, unlike their study which also showed significant changes in visuospatial and language functions over time, such changes were not evident in our study suggesting that such changes were limited in early HD and not across different stages of HD. Executive function domain has always been recognized to be the most vulnerable in HD [34] with progressive impairment evident not just in early stages of HD [32,35] but also in pre-manifest HD patients [36].

In contrast to the HD group, which had minimal change in overall global cognition over time, the control group had a significant improvement in their scores over time. This suggested that controls had in general benefitted from practice effect on repeated testing of measures in the comprehensive neuropsychological test battery. This was consistent to previous works on cognitive performance in healthy controls in longitudinal studies [37,38]. Practice effect in healthy controls is most apparent in the early phases of repetitive testing, with performance scores tending to plateau on subsequent testing [39,40], or after changing to low frequency testing [41]. Atrophy of the caudate nucleus, a structure involved in learning process, is found in normal aging process but this process when compared to healthy controls, occurs at an expedited rate in HD patients as demonstrated through serial radio-imaging studies [31,42]. Nevertheless, as already reported in a 2 to 4 year longitudinal study on cognition in early HD patients, practice effect was evident in certain executive function and memory related tests between first and second assessments [32]. Although it was reported in that same study [32] that practice effect was not observed in language performance, our HD cohort had actually shown a significant improvement in language domain score after 12 months. These findings suggested that HD patients indeed could benefit from practice effect over a relative short time interval and also showed that underlying disease progression may not be translated to measurable cognitive performance changes over short time interval.

Our findings reaffirmed the general slow progression of cognitive deterioration in HD patients over short time interval which inevitably create great difficulty in monitoring cognitive changes in HD patients on routine follow-up in clinic settings. Longitudinal monitoring of disease progression is generally conducted to evaluate potential interventions for delaying phenoconversion in HD thus the generally accepted view is that it is more meaningful to serially evaluate disease progression of pre-manifest HD patients. However, understanding short-term changes and the utility of various cognitive tools in manifest HD patients are also important for multi-disciplinary health team in planning and modifying disease management plans which consists of currently available pharmacological and non-pharmacological interventions aimed at improving patient’s quality of life. Nevertheless, our findings have implications for clinical practice and research. Cognitive decline in HD appeared to be specific in executive function and learning and memory domains after 12 months. Therefore in the clinic, cognitive deterioration in HD over 12-month should not be determined by changes in overall global cognitive score of comprehensive neuropsychological test battery but by detailed analysis of cognitive domain-specific performance. Due to practice effect, it is important in short to medium term longitudinal clinical research to include a control group when assessing the cognition of HD patients.

### Usefulness of brief cognitive tests for longitudinal assessment

As expected, the MMSE, MoCA and the UHDRS cognitive component scores correlated well with overall global cognition, as determined through the comprehensive neuropsychological test battery, in the HD group. These findings support the utility of the three brief cognitive assessment tools in cross-sectional detection of cognitive deficits in manifest HD patients. Furthermore, our findings showed that there were no significant differences between the three brief cognitive tests in reflecting overall global cognition in HD patients, providing no evidence that one test is better than the other in this respect.

However, the baseline scores of comprehensive neuropsychological test battery (overall global cognition) and UHDRS cognitive assessment were highly correlated with their 12-month scores and as judged by the 95% CI of *R*^*2*^ values, both types of assessment had minimal deviations from the linear fit indicated that both tests were reliable and had low score variability over time. The reliability of the MMSE in the HD group, though reasonable, was significantly lower than that for the full neuropsychological test battery and the UHDRS cognitive component. Deficiencies in reliability of MMSE were highlighted in a study by Bowie et al. [43], which inferred that the test was inadequate in detecting small cognitive changes. Moreover, large score variance on annual assessment was another weakness of MMSE as shown in a study on patients with Alzheimer’s disease [44], which further limits its value in assessing disease progression. Similarly in our HD sample, the two versions of MMSE were found to have greater score variance than the comprehensive neuropsychological test battery and the UHDRS cognitive component. Even though the present study demonstrated that there were significant within-group changes after a 12-month period in the MMSE (with ‘World’ spelled backwards) in our HD patients, its use in routine follow-up in clinical practice should be interpreted with caution because of its tendency to vary from one assessment to the next. On the contrary, the MoCA and UHDRS cognitive component, as judged by the differences of *R*^*2*^ values from linear fit models using bootstrap procedure, had comparable performance to the comprehensive neuropsychological battery. Such findings are likely to be attributed by the nature of short-term cognitive progression in HD which is specific to executive function and also the overall design of the tests. The MoCA, which was claimed to have superior sensitivity for detecting MCI compared to MMSE, contains more demanding tasks for assessing executive and memory functions [45] while the UHDRS cognitive component essentially assesses the executive function domain. However, MoCA is an assessment tool that examines multiple cognitive domains hence similar to the overall global cognitive score of comprehensive neuropsychological battery, short-term cognitive decline in HD patients could be masked by practice effect in other domains within the test.

On the basis of high correlation to comprehensive neuropsychological battery and low variance across time, the UHDRS cognitive component is a good brief substitute for comprehensive neuropsychological testing and a sensitive cognitive measure to assess short-term cognitive changes in HD patients compared to MMSE and MoCA. However, the MoCA and MMSE, in that order, might be considered as reasonable alternatives to the ‘gold standard’ for use in clinic setting in circumstances where the UHDRS cognitive component is unavailable but secondary to the limitations of MMSE and MoCA, their results shall be interpreted discretely.

### Other disease measures

There was no significant worsening in the UHDRS behavioural score within our HD group at follow-up, similar to prior observations [18]. Behavioural abnormalities in HD are heterogeneous in nature and without clear temporal progression [46]. Furthermore, psychiatric interventions are often effective in managing behavioural disturbances of HD patients [47] so such features are less likely to exhibit progressive deterioration over time. Thus, the UHDRS behavioural index is not particularly useful as a measure of short to medium term disease progression in HD. In contrast to the absence of measurable change in the behavioural measure, there was a significant increase in the UHDRS motor score over 12 months. This is consistent with the Huntington Study Group’s [18] report of an average three points increase in motor score over six months in manifest HD patients. The ability to demonstrate increase in the UHDRS motor score is not exclusive to manifest HD patients, with another study on pre-manifest patients showing that while the change was minimal after one year, there was significant increase in motor scores over five years [48]. These observations combined suggest that motor deterioration is possibly more aggressive in the short term than cognitive and behavioural changes in HD patients. However, the interpretation of the study findings was undoubtedly constrained by its small sample size and also limited number of patients in different disease stages.

### Concluding remarks

Although MMSE and MoCA have been evaluated in previous cross-sectional HD studies [14-16], the utility of these brief cognitive tests has not been appraised longitudinally. This study provided a new perspective on the utility of two widely used brief cognitive assessment tools (MMSE and MoCA) in comparison to UHDRS cognitive assessment and other measures for monitoring cognitive changes in manifest HD patients over a 12-month period. MMSE and MoCA may be effective for describing global cognition in HD patients in cross-sectional analysis but they are less useful for monitoring longitudinal cognitive changes over short time interval. The UHDRS cognitive assessment, which focuses on testing executive function, is sensitive to short-term cognitive changes in HD and a more reliable brief assessment tool compared to MMSE and MoCA over 12 months. Nevertheless, our findings on the utility of these assessment tools in a restricted cohort of HD patients should be interpreted discretely and further studies on these brief cognitive tests are warranted in the future.

## Abbreviations

HD: Huntington’s disease; MCI: Mild cognitive impairment; MMSE: Mini Mental State Examination; MoCA: Montreal Cognitive Assessment; SD: standard deviation; UHDRS: Unified Huntington’s Disease Rating Scale; (95% CI): 95% confidence interval.

## Competing interests

The authors declare that there are no conflicts of interest in this research.

## Authors’ contributions

The study was conceptualised by EAT, MRM, JDA and TJA. EAT was the main author and analyzed the data, MRM evaluated the manuscript structure, ideas and science, JDA reviewed the manuscript structure, ideas and science, DJM was the main advisor for statistical analyses and appraised the manuscript structure and ideas, LL reviewed manuscript ideas, ADM reviewed the manuscript, TJA evaluated the manuscript structure, ideas and science. The final manuscript was read and approved by all authors.

## Supplementary Material

Additional file 1: Figure S1 Change in neuropsychological battery component tests scores over 12 months. Baseline and 12-month scores for control and HD groups in overall executive domain score (Overall: Ex); letter fluency (LF); action fluency (AF); category switching (CS); Trail Making Test – Part B (Trl.B); Stroop-Interference test (Strp.I); overall working memory domain (Overall: WM); digit forward, backward and sequencing combined score (DF.B.Sq); digit backward (DB); digit sequencing (Dsq); Symbol Digit Modalities Test (SDMT); Ruff 2 &7 Cancellation Test – Accuracy (R27.TotA); overall learning memory & attention domain score (Overall: LM); CVLT - Recall score (CV.Recall); CVLT - Long delayed score (CV.LongD); BVMT – Learning score (BVMT.Learn); BVMT – Delayed recall score (BVMT.Delayd); overall processing speed domain score (Overall: PS); Stroop –Reading test (Strp.WR); Stroop – Naming test (Strp.CN); Trail Making Test – Part A (Trl.A); Ruff 2 & 7 Cancellation Test – Speed (R27.TotS); overall visuospatial domain score (Overall: Vs); Judgement of line (JOL); Rey complex figure copying test (RCF.Copy); overall language domain score (Overall: La); Brief Boston Naming Test (BNT); and Indiana University Token Test (IUTT). Group mean and SD are shown. Click here for file

Additional file 2: Table S1 Scores (mean, SD & range) in control and HD groups at baseline and 12-month. Neuropsychological tests raw scores were converted to standard z-score using test-specific norms. Overall global cognition and domain scores were shown in z-scores. MMSE and MoCA were scored out of 30 points. UHDRS motor and behavioural components were scored in points while individual tests within the cognitive component were reported in z-scores. Click here for file

## References

[B1] Huntington Disease Collaborative Research Group A novel gene containing a trinucleotide repeat that is expanded and unstable on Huntington's disease chromosomes. The Huntington's Disease Collaborative Research Group Cell 1993 72 971 983 8458085 10.1016/0092-8674(93)90585-e

[B2] Blackmore L Simpson SA Crawford JR Cognitive performance in UK sample of presymptomatic people carrying the gene for Huntington's disease J Med Genet 1995 32 358 362 7616542 10.1136/jmg.32.5.358 PMC1050430

[B3] Duff K Paulsen J Mills J Beglinger LJ Moser D Smith MM Langbehn D Stout J Queller S Harrington DL Mild cognitive impairment in prediagnosed Huntington disease Neurology 2010 75 500 507 20610833 10.1212/WNL.0b013e3181eccfa2 PMC2918475

[B4] Kirkwood SC Siemers E Stout JC Hodes ME Conneally PM Christian JC Foroud T Longitudinal cognitive and motor changes among presymptomatic Huntington disease gene carriers Arch Neurol 1999 56 563 568 10328251 10.1001/archneur.56.5.563

[B5] Peavy GM Jacobson MW Goldstein JL Hamilton JM Kane A Gamst AC Lessig SL Lee JC Corey-Bloom J Cognitive and functional decline in Huntington's disease: dementia criteria revisited Mov Disord 2010 25 1163 1169 20629124 10.1002/mds.22953 PMC2910142

[B6] Bates G Harper PS Jones L Huntington's disease 2002 New York: Oxford University Press

[B7] Chodosh J Petitti DB Elliott M Hays RD Crooks VC Reuben DB Galen Buckwalter J Wenger N Physician recognition of cognitive impairment: evaluating the need for improvement J Am Geriatr Soc 2004 52 1051 1059 15209641 10.1111/j.1532-5415.2004.52301.x

[B8] Folstein MF Folstein SE McHugh PR "Mini-mental state". A practical method for grading the cognitive state of patients for the clinician J Psychiatr Res 1975 12 189 198 1202204 10.1016/0022-3956(75)90026-6

[B9] Tombaugh TN McIntyre NJ The mini-mental state examination: a comprehensive review J Am Geriatr Soc 1992 40 922 935 1512391 10.1111/j.1532-5415.1992.tb01992.x

[B10] Godefroy O Fickl A Roussel M Auribault C Bugnicourt JM Lamy C Canaple S Petitnicolas G Is the Montreal Cognitive Assessment Superior to the Mini-Mental State Examination to Detect Poststroke Cognitive Impairment?: A Study With Neuropsychological Evaluation Stroke 2011 42 1712 1716 21474808 10.1161/STROKEAHA.110.606277

[B11] Scazufca M Almeida OP Vallada HP Tasse WA Menezes PR Limitations of the Mini-Mental State Examination for screening dementia in a community with low socioeconomic status: results from the Sao Paulo Ageing & Health Study Eur Arch Psychiatry Clin Neurosci 2009 259 8 15 18560791 10.1007/s00406-008-0827-6

[B12] Wind AW Schellevis FG Van Staveren G Scholten RP Jonker C Van Eijk JT Limitations of the Mini-Mental State Examination in diagnosing dementia in general practice Int J Geriatr Psychiatry 1997 12 101 108 9050431 10.1002/(sici)1099-1166(199701)12:1<101::aid-gps469>3.0.co;2-r

[B13] Nasreddine ZS Phillips NA Bedirian V Charbonneau S Whitehead V Collin I Cummings JL Chertkow H The Montreal Cognitive Assessment, MoCA: a brief screening tool for mild cognitive impairment J Am Geriatr Soc 2005 53 695 699 15817019 10.1111/j.1532-5415.2005.53221.x

[B14] Gluhm S Goldstein J Brown D Van Liew C Gilbert PE Corey-Bloom J Usefulness of the Montreal Cognitive Assessment (MoCA) in Huntington's disease Mov Disord 2013 28 1744 1747 23798501 10.1002/mds.25578 PMC4347861

[B15] Videnovic A Bernard B Fan W Jaglin J Leurgans S Shannon KM The Montreal Cognitive Assessment as a screening tool for cognitive dysfunction in Huntington's disease Mov Disord 2010 25 401 404 20108371 10.1002/mds.22748

[B16] Mickes L Jacobson M Peavy G Wixted JT Lessig S Goldstein JL Corey-Bloom J A comparison of two brief screening measures of cognitive impairment in Huntington's disease Mov Disord 2010 25 2229 2233 20721924 10.1002/mds.23181

[B17] Bezdicek O Majerova V Novak M Nikolai T Ruzicka E Roth J Validity of the Montreal Cognitive Assessment in the detection of cognitive dysfunction in Huntington's disease Appl Neuropsychol Adult 2013 20 33 40 23373682 10.1080/09084282.2012.670158

[B18] Huntington Study Group Unified Huntington's disease rating scale: Reliability and consistency Movement Disorders 1996 11 136 142 8684382 10.1002/mds.870110204

[B19] Sheridan LK Fitzgerald HE Adams KM Nigg JT Martel MM Puttler LI Wong MM Zucker RA Normative Symbol Digit Modalities Test performance in a community-based sample Arch Clin Neuropsychol 2006 21 23 28 16139470 10.1016/j.acn.2005.07.003

[B20] O'Bryant SE O'Jile JR Attenuating Demographic Influences on Verbal Fluency and Animal Naming in a Psychiatric Sample Applied Neuropsychology 2004 11 208 212 10.1207/s15324826an1104_6 15673493

[B21] Swanson J The Delis-Kaplan Executive Function System: A Review Canadian Journal of School Psychology 2005 20 117 128

[B22] Wechsler D Wechsler Adult Intelligence Scale - Fourth Edition (WAIS-IV) administration and scoring manual 2008 San Antonio, TX: Psychological Corporation

[B23] Smith A Symbol Digit Modalities Test Manual 2007 Western Psychological Services: Torrance, CA

[B24] Ruff RM Niemann H Allen CC Farrow CE Wylie T The Ruff 2 and 7 Selective Attention Test: a neuropsychological application Percept Mot Skills 1992 75 1311 1319 1484803 10.2466/pms.1992.75.3f.1311

[B25] Graves RE Bezeau SC Fogarty J Blair R Boston naming test short forms: a comparison of previous forms with new item response theory based forms J Clin Exp Neuropsychol 2004 26 891 902 15742540 10.1080/13803390490510716

[B26] Unverzagt FW Morgan OS Thesiger CH Eldemire DA Luseko J Pokuri S Hui SL Hall KS Hendrie HC Clinical utility of CERAD neuropsychological battery in elderly Jamaicans J Int Neuropsychol Soc 1999 5 255 259 10217925 10.1017/s1355617799003082

[B27] Dalrymple-Alford JC Livingston L MacAskill MR Graham C Melzer TR Porter RJ Watts R Anderson TJ Characterizing mild cognitive impairment in Parkinson's disease Mov Disord 2011 26 629 636 21287603 10.1002/mds.23592

[B28] Gelman A Hill J Data analysis using regression and multilevel/hierarchical models 2007 New York: Cambridge University Press

[B29] Efron B Bootstrap Methods: Another Look at the Jackknife The Annals of Statistics 1979 7 1 26

[B30] Tabrizi SJ Scahill RI Durr A Roos RAC Leavitt BR Jones R Landwehrmeyer GB Fox NC Johnson H Hicks SL Kennard C Craufurd D Frost C Langbehn DR Reilmann R Stout JC Biological and clinical changes in premanifest and early stage Huntington's disease in the TRACK-HD study: the 12-month longitudinal analysis The Lancet Neurology 2010 10 31 42 21130037 10.1016/S1474-4422(10)70276-3

[B31] Tabrizi SJ Reilmann R Roos RA Durr A Leavitt B Owen G Jones R Johnson H Craufurd D Hicks SL Kennard C Landwehrmeyer B Stout JC Borowsky B Scahill RI Frost C Langbehn DR Potential endpoints for clinical trials in premanifest and early Huntington's disease in the TRACK-HD study: analysis of 24 month observational data Lancet Neurol 2012 11 42 53 22137354 10.1016/S1474-4422(11)70263-0

[B32] Bachoud-Levi AC Maison P Bartolomeo P Boisse MF Dalla Barba G Ergis AM Baudic S Degos JD Cesaro P Peschanski M Retest effects and cognitive decline in longitudinal follow-up of patients with early HD Neurology 2001 56 1052 1058 11320178 10.1212/wnl.56.8.1052

[B33] Snowden J Craufurd D Griffiths H Thompson J Neary D Longitudinal evaluation of cognitive disorder in Huntington's disease J Int Neuropsychol Soc 2001 7 33 44 11253840 10.1017/s1355617701711046

[B34] Lawrence AD Sahakian BJ Hodges JR Rosser AE Lange KW Robbins TW Executive and mnemonic functions in early Huntington's disease Brain 1996 119 Pt 5 1633 1645 8931586 10.1093/brain/119.5.1633

[B35] Ho AK Sahakian BJ Brown RG Barker RA Hodges JR Ane MN Snowden J Thompson J Esmonde T Gentry R Moore JW Bodner T Profile of cognitive progression in early Huntington's disease Neurology 2003 61 1702 1706 14694033 10.1212/01.wnl.0000098878.47789.bd

[B36] Lemiere J Decruyenaere M Evers-Kiebooms G Vandenbussche E Dom R Cognitive changes in patients with Huntington's disease (HD) and asymptomatic carriers of the HD mutation–a longitudinal follow-up study J Neurol 2004 251 935 942 15316797 10.1007/s00415-004-0461-9

[B37] McCaffrey RJ Westervelt HJ Issues associated with repeated neuropsychological assessments Neuropsychol Rev 1995 5 203 221 8653109 10.1007/BF02214762

[B38] Salthouse TA Tucker-Drob EM Implications of short-term retest effects for the interpretation of longitudinal change Neuropsychology 2008 22 800 811 18999354 10.1037/a0013091 PMC2593909

[B39] Collie A Maruff P Darby DG McStephen M The effects of practice on the cognitive test performance of neurologically normal individuals assessed at brief test-retest intervals J Int Neuropsychol Soc 2003 9 419 428 12666766 10.1017/S1355617703930074

[B40] Falleti MG Maruff P Collie A Darby DG Practice effects associated with the repeated assessment of cognitive function using the CogState battery at 10-minute, one week and one month test-retest intervals J Clin Exp Neuropsychol 2006 28 1095 1112 16840238 10.1080/13803390500205718

[B41] Bartels C Wegrzyn M Wiedl A Ackermann V Ehrenreich H Practice effects in healthy adults: a longitudinal study on frequent repetitive cognitive testing BMC Neurosci 2010 11 118 20846444 10.1186/1471-2202-11-118 PMC2955045

[B42] Roth J Klempii J Jech R Zidovska J Uhrova T Doubek P Ulmanova O Brozova H Volfova M Serranova T Ruzicka E Caudate nucleus atrophy in Huntington's disease and its relationship with clinical and genetic parameters Funct Neurol 2005 20 127 130 16324236

[B43] Bowie P Branton T Holmes J Should the Mini Mental State Examination be used to monitor dementia treatments? The Lancet 1999 354 1527 1528 10.1016/S0140-6736(99)03486-8 10551506

[B44] Clark CM Sheppard L Fillenbaum GG Galasko D Morris JC Koss E Mohs R Heyman A Variability in annual Mini-Mental State Examination score in patients with probable Alzheimer disease: a clinical perspective of data from the Consortium to Establish a Registry for Alzheimer's Disease Arch Neurol 1999 56 857 862 10404988 10.1001/archneur.56.7.857

[B45] Larner AJ Julayanont P Phillips N Chertkow H Nasreddine Z Montreal Cognitive Assessment (MoCA): Concept and Clinical Review Cognitive Screening Instruments 2013 London: Springer 111 151

[B46] Jauhar S Ritchie S Psychiatric and behavioural manifestations of Huntington's disease Advances in Psychiatric Treatment 2010 16 168 175

[B47] Phillips W Shannon KM Barker RA The current clinical management of Huntington's disease Mov Disord 2008 23 1491 1504 18581443 10.1002/mds.21971

[B48] Rao AK Gordon AM Marder KS Coordination of fingertip forces during precision grip in premanifest Huntington's disease Mov Disord 2011 26 862 869 21394785 10.1002/mds.23606 PMC3116202

