# Supplementary figures and images for: Comparison of cognitive and UHDRS measures in monitoring disease progression in Huntington’s disease: a 12-month longitudinal study
Source: Transl Neurodegener. 2014 Jul 12;3:15. doi: 10.1186/2047-9158-3-15 (PMC4105864; doi:10.1186/2047-9158-3-15)

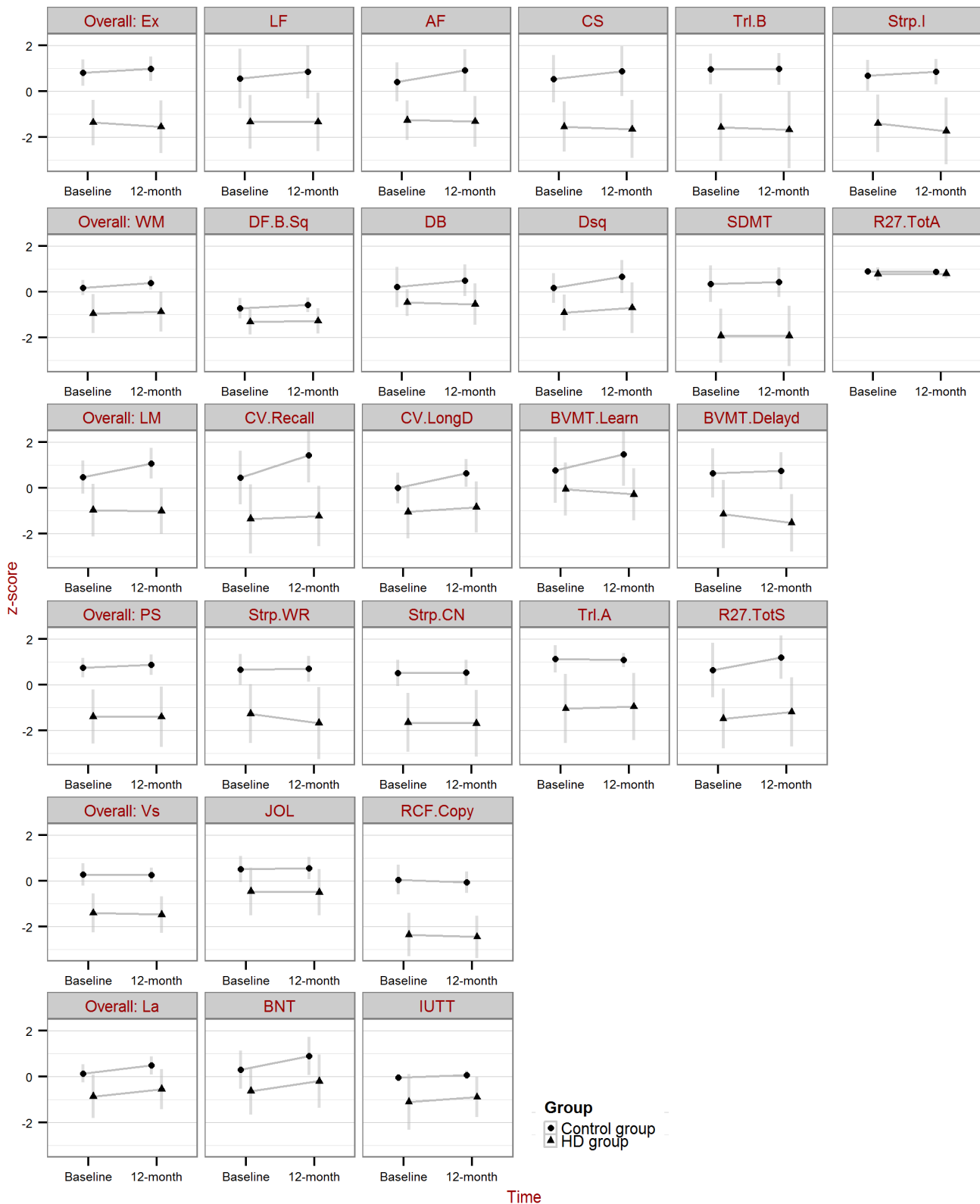

Supplement: Additional file 1: Figure S1 — Change in neuropsychological battery component tests scores over 12 months. Baseline and 12-month scores for control and HD groups in overall executive domain score (Overall: Ex); letter fluency (LF); action fluency (AF); category switching (CS); Trail Making Test – Part B (Trl.B); Stroop-Interference test (Strp.I); overall working memory domain (Overall: WM); digit forward, backward and sequencing combined score (DF.B.Sq); digit backward (DB); digit sequencing (Dsq); Symbol Digit Modalities Test (SDMT); Ruff 2 &7 Cancellation Test – Accuracy (R27.TotA); overall learning memory & attention domain score (Overall: LM); CVLT - Recall score (CV.Recall); CVLT - Long delayed score (CV.LongD); BVMT – Learning score (BVMT.Learn); BVMT – Delayed recall score (BVMT.Delayd); overall processing speed domain score (Overall: PS); Stroop –Reading test (Strp.WR); Stroop – Naming test (Strp.CN); Trail Making Test – Part A (Trl.A); Ruff 2 & 7 Cancellation Test – Speed (R27.TotS); overall visuospatial domain score (Overall: Vs); Judgement of line (JOL); Rey complex figure copying test (RCF.Copy); overall language domain score (Overall: La); Brief Boston Naming Test (BNT); and Indiana University Token Test (IUTT). Group mean and SD are shown. [file 2047-9158-3-15-S1.pdf]
