# Supplementary material for: Comparison of cognitive and UHDRS measures in monitoring disease progression in Huntington’s disease: a 12-month longitudinal study
Source: Transl Neurodegener. 2014 Jul 12;3:15. doi: 10.1186/2047-9158-3-15 (PMC4105864; doi:10.1186/2047-9158-3-15)
Supplement: Additional file 2: Table S1 — Scores (mean, SD & range) in control and HD groups at baseline and 12-month. Neuropsychological tests raw scores were converted to standard z-score using test-specific norms. Overall global cognition and domain scores were shown in z-scores. MMSE and MoCA were scored out of 30 points. UHDRS motor and behavioural components were scored in points while individual tests within the cognitive component were reported in z-scores. [file 2047-9158-3-15-S2.doc]

**Supplementary Table A. Scores (mean, SD & range) in control and HD groups at baseline and 12-month.** Neuropsychological tests raw scores were converted to standard z-score using test-specific norms. Overall global cognition and domain scores were shown in z-scores. MMSE and MoCA were scored out of 30 points. UHDRS motor and behavioural components were scored in points while individual tests within the cognitive component were reported in z-scores

| Measures | Control group | | | |  | HD group | | | |
| --- | --- | --- | --- | --- | --- | --- | --- | --- | --- |
|  | Baseline  Mean (SD) | Range | 12-month  Mean (SD) | Range |  | Baseline  Mean (SD) | Range | 12-month  Mean (SD) | Range |
| Overall global cognition (z-score)  Executive function (z-score)  Working memory & attention (z-score)  Learning & memory (z-score)  Visuospatial (z-score)  Language (z-score)  Processing speed (z-score) | 0.4 (0.3)  0.8 (0.6)  0.2 (0.3)  0.5 (0.7)  0.3 (0.5)  0.1 (0.4)  0.7 (0.4) | -0.2 – 1.1  -0.3 – 2.1  -0.5 – 0.7  -0.8 – 2.0  -0.7 – 0.9  -0.4 – 0.8  -0.1 – 1.8 | 0.7 (0.3)  1.0 (0.5)  0.4 (0.3)  1.1 (0.7)  0.3 (0.3)  0.5 (0.4)  0.9 (0.4) | 1. – 1.2   0.1 – 1.9  -0.0 – 0.9  -0.2 – 2.2  -0.3 – 0.8  -0.2 – 0.8  0.2 – 1.7 |  | -1.2 (0.9)  -1.4 (1.0)  -1.0 (0.8)  -1.0 (1.2)  -1.4 (0.8)  -0.9 (0.9)  -1.4 (1.2) | -2.7 – 0.2  -2.9 – 0.5  -3.0 – 0.2  -2.8 – 1.5  -2.8 – 0.9  -2.5 – 0.8  -3.0 – 0.8 | -1.1 (0.9)  -1.5 (1.1)  -0.9 (0.9)  -1.0 (1.0)  -1.5 (0.8)  -0.5 (0.9)  -1.4 (1.3) | -2.7 – 0.5  -3.0 – 0.8  -3.0 – 0.5  -2.8 – 0.8  -2.8 – 0.1  -2.1 – 0.8  -3.0 – 1.2 |
| MMSE (point)  with WORLD item  with Sevens item | 29.0 (1.1)  28.9 (1.2) | 26 – 30  26 – 30 | 29.5 (0.5)  29.4 (0.8) | 29 – 30  28 – 30 |  | 26.5 (3.2)  24.8 (4.1) | 19 – 30  17 – 30 | 25.7 (3.6)  24.4 (4.0) | 17 – 30  17 – 30 |
| MoCA (point) | 27.8 (1.4) | 24 – 30 | 28.3 (1.6) | 24 – 30 |  | 21.5 (4.9) | 11 – 28 | 21.0 (5.8) | 10 – 30 |
| UHDRS  Motor component* (point)  Cognitive component (z-score)  Letter fluency (z-score)  SDMT (z-score)  Stroop Reading (z-score)  Stroop Naming (z-score)  Stroop Interference (z-score)  Behavioural component* (point) | -  0.6 (0.5)  0.6 (1.3)  0.4 (0.8)  0.7 (0.7)  0.5 (0.6)  0.7 (0.7)  - | -  -0.4 – 1.7  -2.0 – 3.0  -0.8 – 1.7  -1.0 – 1.5  -0.7 – 1.5  -0.7 – 1.5  - | -  0.7 (0.5)  0.9 (1.2)  0.4 (0.8)  0.7 (0.6)  0.5 (0.6)  0.9 (0.6)  - | -  -0.3 – 1.5  -0.7 – 3.0  -0.6 – 1.7  -0.7 – 1.7  -0.7 – 1.3  -0.3 – 1.7  - |  | 42.3 (19.9)  -1.5 (1.1)  -1.3 (1.2)  -1.9 (1.2)  -1.3 (1.3)  -1.6 (1.3)  -1.4 (1.3)  23.4 (14.9) | 11 – 82  -2.9 – 0.8  -3.0 – 1.0  -3.0 – 1.0  -3.0 – 0.5  -3.0 – 0.5  -3.0 – 1.0  0 – 48 | 49.6 (20.4)  -1.7 (1.3)  -1.3 (1.3)  -1.9 (1.3)  -1.7 (1.6)  -1.7 (1.5)  -1.7 (1.5)  24.0 (15.6) | 14 – 80  -3.0 – 1.1  -3.0 – 1.7  -3.0 – 1.2  -3.0 – 1.7  -3.0 – 1.0  -3.0 – 1.3  3 – 49 |

* UHDRS motor and behavioural components were not assessed in the control group.
